# Supplementary figures and images for: Genomic comparison of diverse Salmonella serovars isolated from swine
Source: PLoS One. 2019 Nov 1;14(11):e0224518. doi: 10.1371/journal.pone.0224518 (PMC6824618; doi:10.1371/journal.pone.0224518)

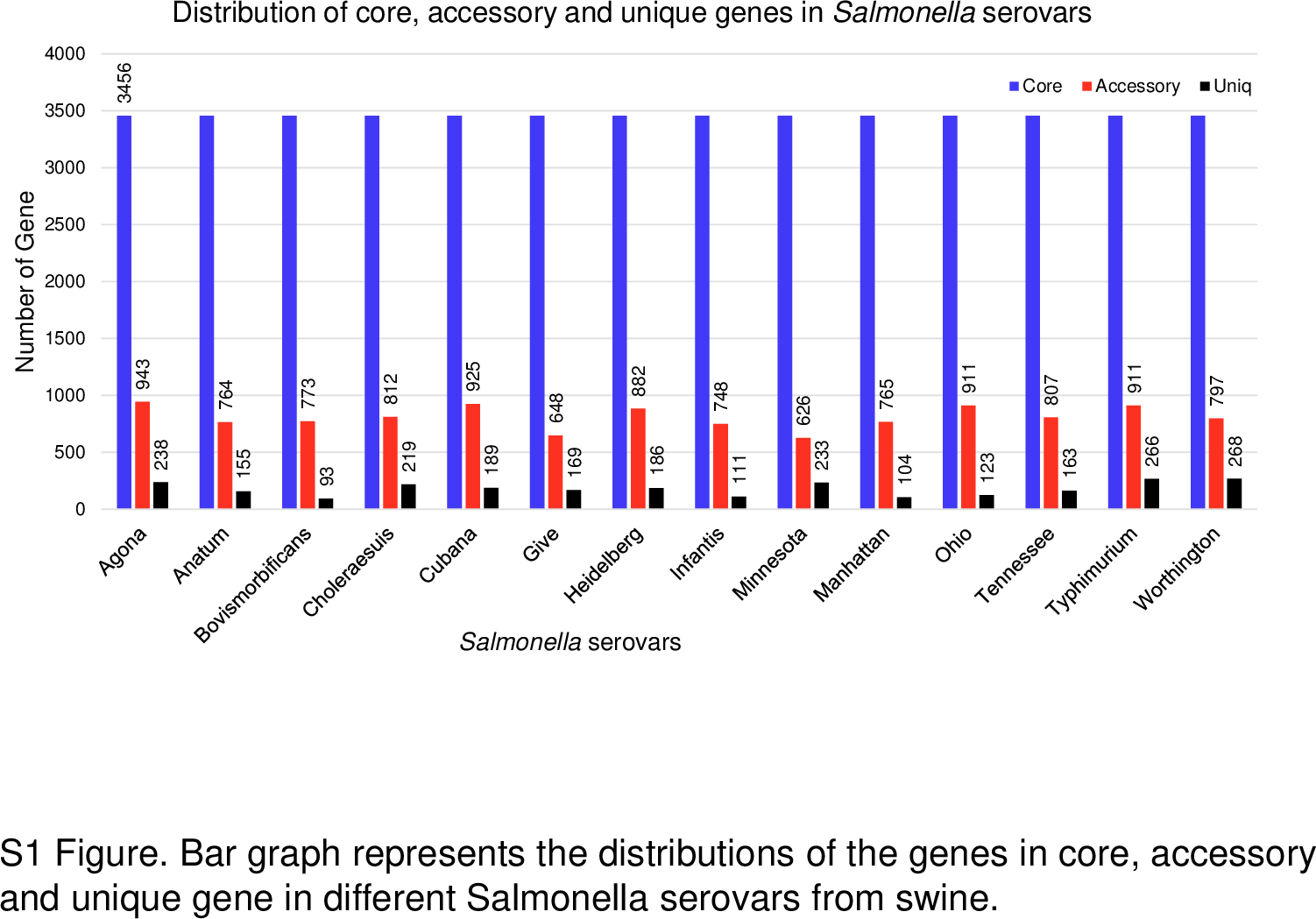

Supplement: S1 Fig — (TIF) [file pone.0224518.s004.tif]
