# Supplementary material for: Genomic comparison of diverse Salmonella serovars isolated from swine
Source: PLoS One. 2019 Nov 1;14(11):e0224518. doi: 10.1371/journal.pone.0224518 (PMC6824618; doi:10.1371/journal.pone.0224518)
Supplement: Attachment — Submitted filename: CommentToAuthorsPlosOne.docx [file pone.0224518.s005.docx]

Gupta et al., examined the genomic profiles of diverse *Salmonella* serovars from swine using whole genome sequencing. Overall there are some interesting pieces, but the paper needs some major revisions and editing. Many of the specific areas of concern or further consideration are provided below.

**Title**

Ln3: Please remove additional ‘+’ symbol after the author name Jonathan G. Frye.

**Abstract**

Ln44: “More than 75% of the genes were part of the core genome”, according to Ln252 it is ~ 42% please confirm.

Ln52: Expand CRISPR.

Ln55: The keywords *Salmonella* and serovar can be removed and add new keywords because these two are appearing in title and arrange the keywords in descending order.

**Introduction**

Ln73: Please remove the” >” symbol because” more than” is already written.

Ln76-78: In general objective and hypothesis are written at the end of the introduction. However, they are written at Ln76-78.

Ln78: were *Salmonella* serovars collected from swine swabs?

**Materials and Methods**

Ln108-110: what are concentrations used for antibiotics panel used? Which solvent did used for solubility?

**Results**

Ln143: “This study was the part of retrospective study” were the samples used in this study obtained from the McMillan et al., 2019 (ref # 22)? It is not clear in M&M section.

Ln152-157: Data not available in Table 1.

Ln156: Expand ‘ANI’.

LN158: Expand ‘SRA’.

Ln164: Expand ‘CLSI’.

Ln172: Please correct the sentence.

Ln177-179: The information related to ‘total number of AR genes’ is not available in Table 1.

Ln178: Please include the total number of genomes before ‘genome sequences’.

Ln179-Ln182: Please recheck the result, the gene variants Y and aa of aac6-I were not found in the table 2.

Ln184: According to table2, only seven isolates were resistance to beta-lactams. Please check.

Ln198: bla_CARB-2_ is 2/14 right?

Ln207-209: Please check the results, the gene “*ereA”* is not found in Agona. Please restructure the sentence.

Ln211: “both “only in Agona both *sul1* and *sul2* were detected but not in *typhimurium* were only *sul1* were detected.

Ln213: Please remove ‘chloramphenicol resistant’ or rearrange the sentence because not all the fourteen are resistance to chloramphenicol.

Ln214-215: What is the need for writing *cml* or *cat* resistance genes here because they are not detected in the study.

Ln215-220: Data not available.

Ln221: How were integrons detected? This information is not available in M & M section.

Ln231: How were plasmids detected? This information is not available in M & M section.

Ln233-237: The replicon types such as *IncII, IncFII, IncN*, *IncP6*, *col440II* and *IncQ* were not found in table 2.

Ln240-241: Please provide the complete legend for Fig.1 Example what does arrows indicated?

Ln252: The total number of unique genes 2,324 is correct? From Fig.2 they are 2,517. Please make changes.

Ln255: Please include how floral diagram is created in the M & M section?

Ln265: Please remove’(ref)’

Ln266: I could see only 22 COG functions in figure 3.

Ln269-270: Suggestions to change Y-axis as percentages and keep only most abundant functions (may be top 10 + combining all other functions).

Ln273: Please include number of genes near “highest and lowest”.

Ln275: Expand ‘E’ completely.

Ln288: Please include exact percentage values for G and L.

Ln293-328: These results are based on supplementary tables and these are presented for each isolate and it is difficult to compare the results. A comprehensive information like table 2 will help to look over the results clearly.

Ln357-359: Fig.5 results were not discussed in results section.

Ln362: This is ten right?

**Discussion**

Discussion is well organized however it is too lengthy, restatement of data (restatement of results) observed in some places.

**References**

Too many references.

**Figures**

Except figure 2, none of the figures are in good quality they are not clearly visible for interpretation, in addition more information is needed to add in the legend for easy interpretation.
