# Supplementary material for: Genomic comparison of diverse Salmonella serovars isolated from swine
Source: PLoS One. 2019 Nov 1;14(11):e0224518. doi: 10.1371/journal.pone.0224518 (PMC6824618; doi:10.1371/journal.pone.0224518)
Supplement: Attachment — Submitted filename: Response to Reviewers.docx [file pone.0224518.s006.docx]

**Reviewer #1:**

Gupta et al., examined the genomic profiles of diverse *Salmonella* serovars from swine using whole genome sequencing. Overall there are some interesting pieces, but the paper needs some major revisions and editing. Many of the specific areas of concern or further consideration are provided below.

**Title**

Ln3: Please remove additional ‘+’ symbol after the author name Jonathan G. Frye.

**Response:** We have deleted the additional + symbol in Ln3 in the revised manuscript.

**Abstract**

Ln44: “More than 75% of the genes were part of the core genome”, according to Ln252 it is ~ 42% please confirm.

**Response:** We would like to thank the reviewer for this observation, the authors want to convey that >75% of the genes in each genome were part of the core genome. To avoid confusion we have corrected the text accordingly in Ln 44.

Ln52: Expand CRISPR.

**Response:** CRISPR was expanded in Ln 52.

Ln55: The keywords *Salmonella* and serovar can be removed and add new keywords because these two are appearing in title and arrange the keywords in descending order.

**Response:** New key words have been added and arranged in descending order in Ln 57 in the revised manuscript.

**Introduction**

Ln73: Please remove the” >” symbol because” more than” is already written.

**Response:** “>” symbol has been deleted in Ln71 in the revised manuscript.

Ln76-78: In general objective and hypothesis are written at the end of the introduction. However, they are written at Ln76-78.

**Response:** We would like to thank the reviewer for this observation. The text has been deleted and added at the end of the introduction (Ln 92-97).

Ln78: were *Salmonella* serovars collected from swine swabs?

**Response:** The Salmonella serovars were from swine swabs.

**Materials and Methods**

Ln108-110: what are concentrations used for antibiotics panel used? Which solvent did used for solubility?

**Response:** All the antibiotic concentrations were as per Clinical and Laboratory Standards Institute (CLSI) recommendations. Minimum inhibitory concentrations (MICs) were manually recorded by SensiTouch®, and CLSI standards were used to determine resistance. The antibiotics were solublized in adequate solvent as per manufacturer’s recommendation.

**Results**

Ln143: “This study was the part of retrospective study” were the samples used in this study obtained from the McMillan et al., 2019 (ref # 22)? It is not clear in M&M section.

**Response**: This is a comprehensive study in which different Salmonella serovars genomes exclusively from swine were analyzed. The isolates were obtained from the previous study.

Ln152-157: Data not available in Table 1.

**Response:** Adequate data has been added in the Table 1 in the revised manuscript.

Ln156: Expand ‘ANI’.

**Response:** ANI has been expanded in the text in Ln155 in the revised manuscript.

LN158: Expand ‘SRA’.

**Response:** SRA has been expanded in the text in Ln157 in the revised manuscript.

Ln164: Expand ‘CLSI’.

**Response:** CLSI has been expanded in the text in Ln164-165 in the revised manuscript.

Ln172: Please correct the sentence.

**Response:** The sentence has been corrected in Ln172-173 in the revised manuscript.

Ln177-179: The information related to ‘total number of AR genes’ is not available in Table 1.

**Response:** The information regarding total number of AR genes has been added in Table 1.

Ln178: Please include the total number of genomes before ‘genome sequences’.

**Response:** We are not clear what the Reviewer is asking here. We sequenced 194 genomes from different food animals out of which 14 genomes from swine were used in this study.

Ln179-Ln182: Please recheck the result, the gene variants Y and aa of aac6-I were not found in the table 2.

**Response:** The chromosomally located cryptic gene aac6-IY and aac6-Iaa were uniformly detected in all the isolates. It does not confer any resistance due to mutation in the promoter region, thus these genes were not included in Table 2.

Ln184: According to table2, only seven isolates were resistance to beta-lactams. Please check.

**Response:** We would like to thank the reviewer for this observation. The text has been corrected in Ln184-185 in the revised manuscript.

Ln198: bla_CARB-2_ is 2/14 right?

**Response:** We would like to thank the reviewer for this comment. The digits has been corrected in Ln199 in the revised manuscript.

Ln207-209: Please check the results, the gene “*ereA”* is not found in Agona. Please restructure the sentence.

**Response:** We would like to thank the reviewer for this comment. The sentence has been restructured in Ln208-209 in the revised manuscript.

Ln211: “both “only in Agona both *sul1* and *sul2* were detected but not in *typhimurium* were only *sul1* were detected.

**Response:** We would like to thank the reviewer for this comment. We have reframed and included the serovars details in Ln210-212 in the revised manuscript.

Ln213: Please remove ‘chloramphenicol resistant’ or rearrange the sentence because not all the fourteen are resistance to chloramphenicol.

**Response:** We would like to thank the reviewer for this comment. We have reframed the sentence in Ln214 in the revised manuscript.

Ln214-215: What is the need for writing *cml* or *cat* resistance genes here because they are not detected in the study.

**Response:** We have deleted *cml* or *cat* resistance genes.

Ln215-220: Data not available.

**Response:** The data has been added as a supplementary table (S2 Table).

Ln221: How were integrons detected? This information is not available in M & M section.

**Response:** We have added the details of integrons detection in Ln137-138 in the revised manuscript.

Ln231: How were plasmids detected? This information is not available in M & M section.

**Response:** We have added the tool used to detect plasmid replicon in Ln139-140 in the revised mansucript.

Ln233-237: The replicon types such as *IncII, IncFII, IncN*, *IncP6*, *col440II* and *IncQ* were not found in table 2.

**Response:** Plasmid replicon information has been updated in Table 2 in the revised manuscript.

Ln240-241: Please provide the complete legend for Fig.1 Example what does arrows indicated?

**Response:** We have added the legends for Figure 1 in the revised manuscript.

Ln252: The total number of unique genes 2,324 is correct? From Fig.2 they are 2,517. Please make changes.

**Response:** We have changed the total number of unique gene in Figure 2 in Ln256-257 in the revised manuscript.

Ln255: Please include how floral diagram is created in the M & M section?

**Response:** We have included the tool used to create floral diagram in the revised manuscript.

Ln265: Please remove’(ref)’

**Response:** We have added the reference in line 265 in the revised manuscript.

Ln266: I could see only 22 COG functions in figure 3.

**Response:** We have given details of 22 cog categories because among all the COG categories, chromatin structure and dynamics (B) category was not present in all the Salmonella serovars.

Ln269-270: Suggestions to change Y-axis as percentages and keep only most abundant functions (may be top 10 + combining all other functions).

**Response:** We have re structured the figure and and included the top most abundant functional group and combined all other functional group as suggested by the Reviewer in the revised manuscript.

Ln273: Please include number of genes near “highest and lowest”.

**Response:** We have included the number of genes in Ln277 as suggested by the Reviewer in the revised manuscript.

Ln275: Expand ‘E’ completely.

**Response:** We have expanded ‘E’ in Ln279 in the revised manuscript.

Ln288: Please include exact percentage values for G and L.

**Response:** The percentage value has been added in Ln 295-296 in the revised manuscript.

Ln293-328: These results are based on supplementary tables and these are presented for each isolate and it is difficult to compare the results. A comprehensive information like table 2 will help to look over the results clearly.

**Response:** All the virulence data table will be too lengthy to include, however, the genes which were not detected in all the serovars are included in Table 2.

Ln357-359: Fig.5 results were not discussed in results section.

**Response:** The combined results were discussed in the results section in Ln349-360.

Ln362: This is ten right?

**Response:** We have detected resistance and virulence genes in ten isolates, but only eight isolates harbored at least one virulence gene on phage.

**Discussion**

Discussion is well organized however it is too lengthy, restatement of data (restatement of results) observed in some places.

**Response:** The authors have restructured the sentences to avoid redundancy and shorten the revised manuscript.

**References**

Too many references.

**Response:** Some of the references have been deleted in the revised manuscript. However, we have still included many as they are important compare the findings in our study to previous work in the literature.

**Figures**

Except figure 2, none of the figures are in good quality they are not clearly visible for interpretation, in addition more information is needed to add in the legend for easy interpretation.

**Response:** We have modified the figures for clarity. We have submitted the high quality figures with 300 DPI as per manuscript requirements.

**Reviewer #2:**

The manuscript reports an extensive genome analysis of 14 diverse Salmonella serotypes isolated from swine using whole genome sequencing. The results suggest that majority of the genes were part of core genome and dispensable genes were acquired for adaptability. Here, the authors have performed analysis using various bioinformatics tools to identify and predict genetic repertoire in salmonella serovars. I found this paper of broad relevance to the field, especially to antibiotic resistance and public health. However, I have provided general comments and suggestions below that are necessary to improve the manuscript.

Minor comments

1. The authors have stated high concordance between phenotypically confirmed resistances and detected ARGs, however they did not state why it was not well correlated. The authors need to explain.

**Response:** We would like to thank the reviewer for this comment. The panel which we have used for phenotypic resistance assay contains only seventeen antimicrobials and does not include trimethoprim, chloramphenicol and fosfomycin. Hence, the authors have not included the genes conferring resistance to these antimicrobials for the correlation analysis. However, we have described rest of the resistance genes and their corresponding phenotypic resistance in the results and discussions section. We noticed high concordance between predicted resistance and detected phenotype.

2. The authors noticed two distinct pattern of CRISPR-cas3 gene, how did they validated the findings?

**Response**: Our analysis is based on the previous studies reporting two different cas 3 genes in Salmonella. We have aligned all the cas3 genes and constructed the phylogenetic tree which clearly clustered these isolates into two different clads.

3. The authors have showed clusters of orthologous genes in core and accessory genes (figure 4). Authors might also consider to show the distribution of cogs in unique genes.

**Response**: We have included the clusters of orthologous genes in unique genes and modified the figure and text accordingly.

4. Authors might consider to add a figure showing the distribution of pan genome (core genes, accessary genes and unique genes) content in each salmonella serotypes (for eg: serovars in x-axis and number of genes in y-axis).

**Response:** We have added the additional supplementary figure (S1 Figure) showing the distribution of pan genome (core genes, accessary genes and unique genes) content in each *Salmonella* serotypes.

5. The authors need to elaborate the materials and methods sections.

**Response:** The materials and methods section have been elaborated for more clarity in the revised manuscript.

6. Authors should add cogs database reference (line 265)

**Response:** The authors would like to thank the reviewer for pointing out this. The COG database reference has been included in Ln269 in the revised manuscript.
